# The key player problem in complex oscillator networks and electric power grids: Resistance centralities identify local vulnerabilities

**DOI:** 10.1126/sciadv.aaw8359

**Published:** 2019-11-22

**Authors:** M. Tyloo, L. Pagnier, P. Jacquod

**Affiliations:** 1 Institute of Physics, École Polytechnique Fédérale de Lausanne (EPFL), CH-1015 Lausanne, Switzerland.; 2 School of Engineering, University of Applied Sciences of Western Switzerland HES-SO, CH-1951 Sion, Switzerland.; 3 Department of Quantum Matter Physics, University of Geneva, CH-1211 Geneva, Switzerland.

## Abstract

Identifying key players in coupled individual systems is a fundamental problem in network theory. We investigate synchronizable network-coupled dynamical systems such as high-voltage electric power grids and coupled oscillators on complex networks. We define key players as nodes that, once perturbed, generate the largest excursion away from synchrony. A spectral decomposition of the coupling matrix gives an elegant solution to this identification problem. We show that, when the coupling matrix is Laplacian, key players are peripheral in the sense of a centrality measure defined from effective resistance distances. For linearly coupled systems, the ranking is efficiently obtained through a single Laplacian matrix inversion, regardless of the operational synchronous state. The resulting ranking index is termed LRank. When nonlinearities are present, a weighted Laplacian matrix inversion gives another ranking index, WLRank. LRank provides a faithful ranking even for well-developed nonlinearities, corresponding to oscillator angle differences up to approximately Δθ ≲ 40°.

## Teaser

Most vulnerable components identified by resistance centralities in electric power grids.

## INTRODUCTION

Because of growing electric power demand, increasing difficulties with building new lines, and the emergence of intermittent new renewable energy sources, electric power systems are more often operated closer to their maximal capacity (*1*, *2*). Accordingly, their operating state, its robustness against potential disturbances, and its local vulnerabilities need to be assessed more frequently and precisely. Furthermore, because electricity markets become more and more integrated, it is necessary to perform these assessments over geographically larger areas. Grid reliability is commonly assessed against *n* − 1 feasibility, transient stability, and voltage stability, by which one means that a grid is considered reliable if (i) it still has an acceptable operating state after any one of its *n* components fails, (ii) that acceptable state is reached from the original state following the transient dynamics generated by the component failure, and (iii) the new operating state is robust against further changes under operating conditions such as changes in power productions and loads. This *n* − 1 contingency assessment is much harder to implement in real-time for a power grid loaded close to its capacity where the differential equations governing its dynamics become nonlinear—the fast, standardly used linear approximation breaks down as the grid is more and more heavily loaded. Nonlinear assessment algorithms have significantly longer runtimes, which makes them of little use for short-time evaluations. In the worst cases, they sometimes even do not converge. Briefly, heavily loaded grids need more frequent and more precise reliability assessments, which are however harder to obtain, precisely because the loads are closer to the grid capacities.

Developing real-time procedures for *n* − 1 contingency assessment requires new, innovative algorithms. One appealing avenue is to optimize contingency ranking (*3*) to try and identify a subset of *n*_s_ < *n* grid components containing all the potentially critical components. The *n* − 1 contingency assessment may then focus on that subset only, with a substantial gain in runtime if *n*_s_ ≪ *n*. Identifying such a subset requires a ranking algorithm for grid components, following some well-chosen criterion. Procedures of this kind have been developed in network models for social and computer sciences, biology, and other fields, in the context of the historical and fundamental problem of identifying the key players (*4*–*8*). They may be, for instance, the players who, once removed, lead to the biggest changes in the other player’s activity in game theory or to the biggest structural change in a social network. That problem has been addressed with the introduction of graph-theoretic centrality measures (*9*, *10*), which order nodes from the most “central” to the most “peripheral”—in a sense that they themselves define. A plethora of centrality indices has been introduced and discussed in the literature on network theory (*9*, *10*), leading up to PageRank (*11*). The latter ranks nodes in a network according to the stationary probability distribution of a Markov chain on the network; accordingly, it gives a meaningful ranking of websites under the reasonable assumption that web surfing is a random process. Their computational efficiency makes PageRank and other purely graph-theoretic indicators very attractive to identify key players on complex networks. It is thus quite tempting to apply purely graph-theoretic methods to identify fast and reliably key players in network-coupled dynamical systems.

Processes such as web crawling for information retrieval are essentially random diffusive walks on a complex network, with no physical conservation law beyond the conservation of probability. The situation is similar for disease (*12*) or rumor (*13*) spreading and for community formation (*14*) where graph-theoretic concepts of index, centrality, betweenness, coreness, and so forth have been successfully applied to identify tightly bound communities.

Coupled dynamical systems such as complex supply networks (*15*), electric power grids (*16*), consensus algorithm networks (*17*), or more generally network-coupled oscillators (*18*, *19*) are, however, fundamentally different. There, the randomness of motion on the network giving, e.g., the Markov chain at the core of PageRank, is replaced by a deterministic dynamics supplemented by physical conservation laws that cannot be neglected. Pure or partially extended graph-theoretic methods have been applied in vulnerability investigations of electric power grids (*20*–*22*) and investigations of cascades of failures in coupled communication and electric power networks (*23*, *24*). They have, however, been partially or totally invalidated by investigations on more precise models of electric power transmission that take fundamental physical laws into account (in this case, Ohm’s and Kirchhoff’s laws) (*25*, *26*). It is therefore doubtful that purely topological graph-theoretic descriptors are able to identify the potentially critical components in deterministic, network-coupled dynamical systems. Purely graph-theoretic approaches need to be extended to account for physical laws (*20*). The influence of the dynamics on transient performance for regular graphs on *d*-dimensional tori has been emphasized in (*27*).

Here, we give an elegant solution to the key player problem for a family of deterministic, network-coupled dynamical systems related to the Kuramoto model (*18*, *19*). While we focus mostly on high-voltage electric power grids whose swing dynamics, under the lossless line approximation, is given by a second-order version of the Kuramoto model (*16*, *28*), we show that our approach also applies to other generic models of network-coupled oscillators. Key players in these systems can be defined in various ways. For instance, they can be identified by an optimal geographical distribution of system parameters such as inertia, damping, or natural frequencies or alternatively as those whose removal leads to the biggest change in operating state. Here, we define key players as those nodes where a local disturbance leads to the largest short-time transient network response. In the context of electric power grids, transient stability is the ability of the grid to maintain synchrony under relatively large disturbances such as loss or fluctuations of power generation or of a large load (*29*). If under such a fault, the system remains in the vicinity of its original state, it has maintained synchrony. There are different measures to quantify the magnitude of the transient excursion, such as nadir and maximal rate of change of the network-averaged frequency (*30*, *31*) or other dynamical quantities such as network susceptibilities (*32*) and the wave dynamics following disturbances (*33*). Here, we quantify the total transient excursion through performance measures that are time-integrated quadratic forms in the system’s degrees of freedom (see Materials and Methods and the Supplementary Materials). Transient excursions typically last 10 to 20 s in large, continental power grids, which sets the time scales we are interested in.

**Fig. 1:**
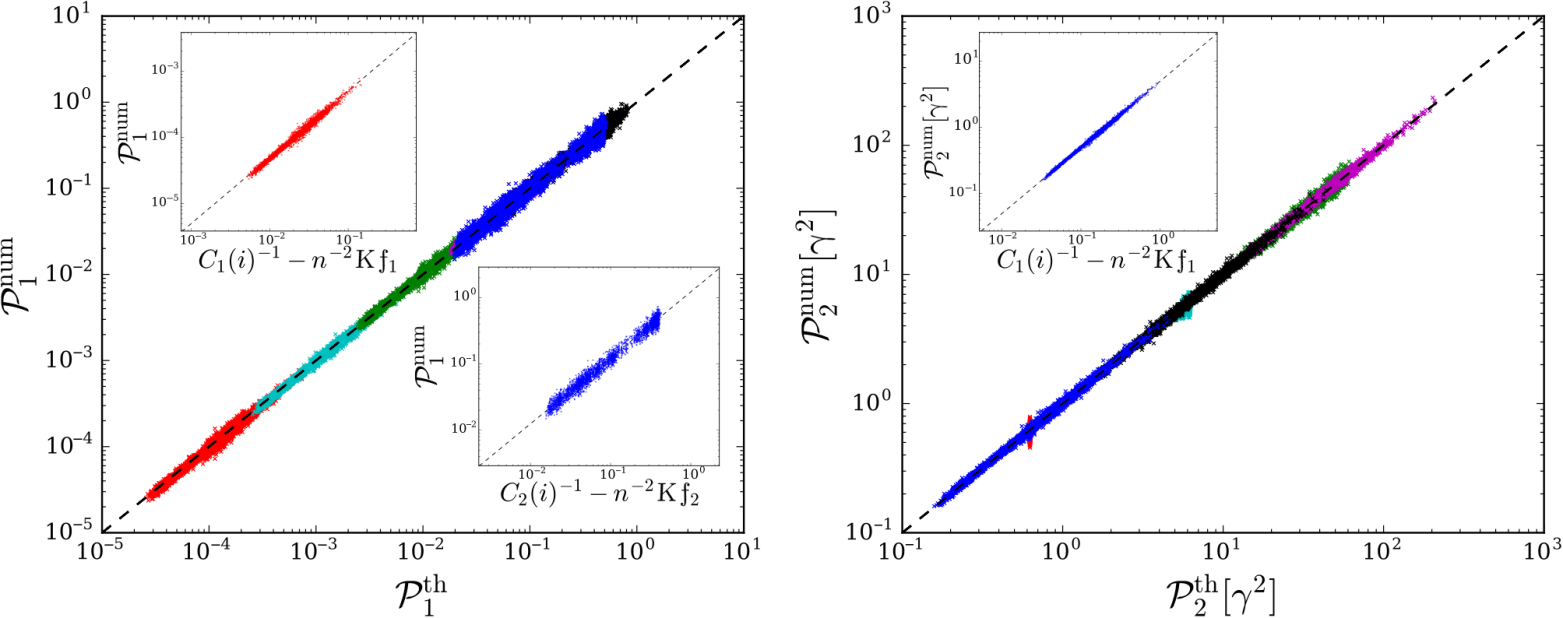
Comparison between theoretical predictions and numerical results for both performance measures $P_{1}$ and $P_{2}$ defined in Eq. 3. Each point corresponds to a noisy disturbance on a single node of the European electric power grid sketched in Fig. 2A (see Materials and Methods and the Supplementary Materials) and governed by Eq. 1 with constant inertia and damping parameters. The time-dependent disturbance δ*P*_*i*_(*t*) is defined by an Ornstein-Uhlenbeck noise of magnitude δ*P*_0_ = 1 and correlation time γτ_0_ = 4 × 10^−5^ (red crosses), 4 × 10^−4^ (cyan), 4 × 10^−3^ (green), 4 × 10^−2^ (purple), 4 × 10^−1^ (black), and 4 (blue). Time scales are defined by the ratio of damping to inertia parameters γ = *d_i_*/*m_i_* = 0.4 *s*^−1^, which is assumed constant with *d_i_* = 0.02 s. The insets show 𝒫_1_ and 𝒫_2_ as a function of the resistance distance–based graph-theoretic predictions of Eq. 6 valid in both limits of very large and very short noise correlation time τ_0_. The limit of short τ_0_ for 𝒫_2_ gives a node-independent result (Eq. 6).

Anticipating on results to come, Fig. 1 illustrates the excellent agreement between analytical theory and numerical calculations for these performance measures. Particularly interesting is that in both asymptotic limits of quickly and slowly decorrelating noisy disturbance, the performance measures are simply expressed in terms of the resistance centrality (*34*, *35*), which is a variation of the closeness centrality (*9*) based on resistance distances (*36*). This is shown in the insets of Fig. 1. Our main finding is that the resistance centrality is the relevant quantity to construct ranking algorithms in network-coupled dynamical systems.

## RESULTS

We consider network-coupled dynamical systems defined by sets of differential equations of the form

$$m_{i}{\ddot{\theta }}_{i}+d_{i}{\dot{\theta }}_{i}=P_{i}-\sum_{j}b_{\mathrm{ij}}\text{sin}\;(\theta_{i}-\theta_{j}),i=1,\ldots ,n$$ (1)

The coupled individual systems are oscillators with a compact angle degree of freedom θ*_i_* ∈ ( − π, π). Their uncoupled dynamics are determined by natural frequencies *P_i_* (*37*), inertia parameters *m_i_*, and damping parameters *d_i_*. Because the degrees of freedom are compact, the coupling between oscillators needs to be a periodic function of angle differences, and here, we only keep its first Fourier term. The coupling between pairs of oscillators is defined on a network whose Laplacian matrix has elements $L_{\mathrm{ij}}^{\left(0\right)}=-b_{\mathrm{ij}}$ if *i* ≠ *j* and $L_{\mathrm{ii}}^{\left(0\right)}=\sum_{k\neq i}b_{\mathrm{ik}}$. Without inertia, *m_i_* = 0 ∀*i*, Eq. 1 gives the celebrated Kuramoto model on a network with edge weights *b_ij_* > 0, ∀*i*, *j* (*18*, *19*). With inertia on certain nodes, it is an approximate model for the swing dynamics of high-voltage electric power grids in the lossless line approximation (*16*, *28*, *38*). The latter is justified in high-voltage transmission grids, where the resistance is smaller than the reactance typically by a factor of 10 or more. Applied to high-voltage grids, Eq. 1 describes the transient behavior of power grids on time scales of up to roughly 10 to 20 s. Over these time intervals, voltage amplitudes of high-voltage power grids are almost constant; accordingly, it is justified to consider only the dynamics of voltage angles (*29*). Here, we are interested in that transient time regime and accordingly focus on the voltage angle dynamics given by Eq. 1. When angle differences are small, a linear approximation sin(θ*_i_* − θ*_j_*) ≃ θ*_i_* − θ*_j_* is justified, giving first-order (without) or second-order (with inertia) consensus dynamics (*17*).

When the natural frequencies *P_i_* are not too large, synchronous solutions exist that satisfy Eq. 1 with ${\ddot{\theta }}_{i}=0$ and ${\dot{\theta }}_{i}=\omega_{0}$, ∀*i*. Without loss of generality, one may consider Eq. 1 in a frame rotating with the synchronous angular frequency ω_0_, in which case, these states correspond to stable fixed points with ${\dot{\theta }}_{i}=0$. We consider a fixed point with angle coordinates $\theta^{\left(0\right)}=(\theta_{1}^{\left(0\right)},\ldots ,\theta_{n}^{\left(0\right)})$ corresponding to natural frequencies $P^{\left(0\right)}=(P_{1}^{\left(0\right)},\ldots ,P_{n}^{\left(0\right)})$, to which we add a time-dependent disturbance, $P_{i}(t)=P_{i}^{\left(0\right)}+\delta P_{i}(t)$. In the case of electric power grids, we will consider fixed points that are solutions to an optimal power flow problem. These solutions account for physical grid constraints such as thermal (i.e., capacity) limits of the lines and technical limitations of the power plants as well as economic constraints following from different production costs for different power plant types (see Materials and Methods and the Supplementary Materials) (*39*). Linearizing the dynamics about that solution, Eq. 1 becomes

$$m_{i}\;\delta {\ddot{\theta }}_{i}+d_{i}\;\delta {\dot{\theta }}_{i}=\delta P_{i}(t)-\sum_{j}b_{\mathrm{ij}}\text{cos}(\theta_{i}^{\left(0\right)}-\theta_{j}^{\left(0\right)})({\delta \theta }_{i}-{\delta \theta }_{j}),i=1,\ldots ,n$$ (2)

where $\delta \theta_{i}(t)=\theta_{i}(t)-\theta_{i}^{\left(0\right)}$. This set of coupled differential equations governs the small signal response of the system corresponding to weak disturbances. The couplings are defined by a weighted Laplacian matrix $L_{\mathrm{ij}}(\theta^{\left(0\right)})=-b_{\mathrm{ij}}\text{cos}(\theta_{i}^{\left(0\right)}-\theta_{j}^{\left(0\right)})$ if *i* ≠ *j* and $L_{\mathrm{ii}}(\theta^{\left(0\right)})=\sum_{k}b_{\mathrm{ik}}\text{cos}(\theta_{i}^{\left(0\right)}-\theta_{k}^{\left(0\right)})$, which contains information about both the topology of the network and the operational state of the system. This weighted Laplacian matrix significantly differs from the network Laplacian $L^{\left(0\right)}$ when angle differences between coupled nodes are large.

We assess the nodal vulnerability of the system defined in Eq. 1 via the magnitude of the transient dynamics determined by Eq. 2 under a time-dependent disturbance δ*P_i_*(*t*). We take the latter as an Ornstein-Uhlenbeck noise on the natural frequency of a single node, with vanishing average, $\bar{\delta P_{i}(t)}=0$, variance $\delta P_{0}^{2}$, and correlation time τ_0_, $\bar{\delta P_{i}(t_{1})\delta P_{j}(t_{2})}=\delta_{\mathrm{ik}}\;\delta_{\mathrm{jk}}\;\delta P_{0}^{2}\text{exp}[-|t_{1}-t_{2}|/\tau_{0}]$. It is sequentially applied on each of the *k* = 1, …, *n* nodes. This noisy test disturbance is designed to investigate network properties on different time scales by varying τ_0_ and identify the set of most vulnerable nodes, i.e., the key players, as the nodes where the system’s response to δ*P_k_*(*t*) is largest. Besides being a probe to test nodal vulnerabilities, these noisy disturbances alternatively model fluctuating renewable energy sources in electric power grids. In this latter case, however, the correlation time τ_0_ is no longer a free parameter and is typically of the order of a minute or more, i.e., larger than any dynamical time scale in the system, as we discuss below. We quantify the magnitude of the response to the disturbance with the following two performance measures (*40*)

$${𝒫}_{1}=\underset{T\to \infty }{\text{lim}}T^{-1}\sum_{i}\int_{0}^{T}\bar{{|{\delta \theta }_{i}(t)-\Delta (t)|}^{2}}\;dt$$ (3A)

$${𝒫}_{2}=\underset{T\to \infty }{\text{lim}}T^{-1}\sum_{i}\int_{0}^{T}\bar{{|\delta {\dot{\theta }}_{i}(t)-\dot{\Delta }(t)|}^{2}}\;dt$$ (3B)

They are similar to quadratic performance measures based on L_2_ or ℋ_2_ norms previously considered in the context of electric power grids, networks of coupled oscillators, or consensus algorithms (*30*, *40*–*46*) but differ from them in two respects. First, here, we subtract the averages Δ(*t*) = *n*^−1^∑*_j_* δθ*_j_*(*t*) and $\dot{\Delta }(t)=n^{-1}\sum_{j}\delta {\dot{\theta }}_{j}(t)$, because the synchronous state does not change under a constant angle shift. Without that subtraction, artificially large performance measures may be obtained, which reflect a constant angle drift of the synchronous operational state but not a large transient excursion. Second, we divide 𝒫_1,2_ by *T* before taking *T* → ∞ because we consider a noisy disturbance that is not limited in time and that would otherwise lead to diverging values of 𝒫_1,2_.

Here, we calculate 𝒫_1,2_ for the network-coupled dynamical system defined in Eq. 1 when (i) both inertia and damping parameters are constant, *m_i_* ≡ *m*_0_ and *d_i_* ≡ *d*_0_, (ii) the inertia vanishes, *m_i_* ≡ 0, (iii) the ratio γ ≡ *d_i_*/*m_i_* is constant, and (iv) both inertia and damping vary independently. In cases (i) to (iii), 𝒫_1,2_ can be analytically expressed in terms of resistance centralities that will be introduced in the next section (see Materials and Methods and the Supplementary Materials). The next paragraphs focus on case (i), following which we present numerical data for case (iv), which illustrate the general applicability of these results for not too short noise correlation time.

The performance measures 𝒫_1,2_ can be computed analytically from Eq. 2 via Laplace transforms (see Materials and Methods and the Supplementary Materials), for homogeneous damping and inertia, i.e., *d_i_* = *d* = γ*m_i_*, ∀*i*. In the two limits of long and short noise correlation time τ_0_, they can be expressed in terms of the resistance centrality of the node *k* on which the noisy disturbance acts and of graph topological indices called generalized Kirchhoff indices (*36*, *40*). Both quantities are based on the resistance distance, which gives the effective resistance Ω*_ij_* between any two nodes *i* and *j* on a fictitious electrical network where each edge is a resistor of magnitude given by the inverse edge weight in the network defined by the weighted Laplacian matrix. One obtains

$$\Omega_{\mathrm{ij}}(\theta^{\left(0\right)})=L_{\mathrm{ii}}^{†}(\theta^{\left(0\right)})+L_{\mathrm{jj}}^{†}(\theta^{\left(0\right)})-L_{\mathrm{ij}}^{†}(\theta^{\left(0\right)})-L_{\mathrm{ji}}^{†}(\theta^{\left(0\right)})$$ (4)

where $L^{†}$ denotes the Moore-Penrose pseudo-inverse of L (*36*). The resistance centrality of the *k*th node is then defined as *C*_1_(*k*) = [*n*^−1^∑*_j_*Ω*_jk_*]^−1^. It measures how central is the node *k*th in the electrical network, in terms of its average resistance distance to all other nodes—more central nodes have smaller *C*_1_(*k*). A network descriptor, the Kirchhoff index, is further defined as (*36*)

$${\text{Kf}}_{1}\equiv \sum_{i<j}\Omega_{\mathrm{ij}}$$ (5)

Generalized Kirchhoff indices Kf*_p_* and resistance centralities *C_p_*(*k*) can be defined analogously from the *p*th power of the weighted Laplacian matrix, which is also a Laplacian matrix (see Materials and Methods and the Supplementary Materials). In terms of these quantities, the performance measures defined in Eq. 3 depend on the value of the noise correlation time τ_0_ relative to the different time scales in the system. The latter are the ratios *d*/λ_α_ of the damping coefficient *d* with the nonzero eigenvalues λ_α_, α = 2, …, *n*, of $L(\theta^{\left(0\right)})$ and the inverse ratio γ^−1^ = *m*/*d* of damping to inertia parameters. In high-voltage power grids, they are approximately given by *d*/λ_α_ < 1 s and *m*/*d* ≅ 2.5 s. Performance measures Eq. 3 can be obtained for any correlation time τ_0_ (see Materials and Methods and the Supplementary Materials). However, it is interesting to consider the specific cases where τ_0_ is the smallest (τ_0_ ≪ *d*/λ_α_, γ^−1^) or the largest (τ_0_ ≫ *d*/λ_α_, γ^−1^, appropriate for noisy power injections from new renewables) time scale in the probed system. The performance measures particularly take the asymptotic values

$${𝒫}_{1}=\{\begin{array}{l} (\delta P_{0}^{2}\tau_{0}/d)(C_{1}^{-1}(k)-n^{-2}{\text{Kf}}_{1}),\;\tau_{0}\ll d/\lambda_{\alpha },\gamma^{-1}\\ \delta P_{0}^{2}(C_{2}^{-1}(k)-n^{-2}{\text{Kf}}_{2}),\;\tau_{0}\gg d/\lambda_{\alpha },\gamma^{-1} \end{array}$$ (6A)

$${𝒫}_{2}=\{\begin{array}{l} (\delta P_{0}^{2}\tau_{0}/\mathrm{dm})(n-1)/n,\;\tau_{0}\ll d/\lambda_{\alpha },\gamma^{-1}\\ (\delta P_{0}^{2}/d\tau_{0})(C_{1}^{-1}(k)-n^{-2}{\text{Kf}}_{1}),\;\tau_{0}\gg d/\lambda_{\alpha },\gamma^{-1} \end{array}$$ (6B)

in the two limits when τ_0_ is the smallest or the largest time scale in the system. After averaging over the location *k* of the disturbed node, $\bar{C_{1,2}^{-1}}=2Kf_{1,2}/n^{2}$, and one recovers the results in (*40*, *42*, *43*) for the global robustness of the system.

These results are remarkable: They show that the magnitude of the transient excursion under a local noisy disturbance is given by either of the generalized resistance centralities *C*_1_(*k*) or *C*_2_(*k*) of the perturbed node and the generalized Kirchhoff indices Kf_1,2_. The latter are global network descriptors and are therefore fixed in a given network with fixed operational state. One concludes that perturbing the less central nodes—those with largest inverse centralities $C_{1,2}^{-1}(k)$—generates the largest transient excursion. In a given network, key players are therefore nodes with smallest resistance centralities. It is important to keep in mind, however, that these centralities correspond to the weighted Laplacian defined above, where internodal couplings are normalized by the cosine of voltage angle differences. Accordingly, these centralities are dependent on the initial operating state. The asymptotic analytical results of Eq. 6 are corroborated by numerical results in the insets of Fig. 1, obtained directly from Eq. 1, i.e., without the linearization of Eq. 2. The validity of the general analytical expressions for any τ_0_ (see Materials and Methods and the Supplementary Materials) is further confirmed in the main panel of Fig. 1 and by further numerical results obtained for different networks shown in Materials and Methods and the Supplementary Materials.

**Fig. 2:**
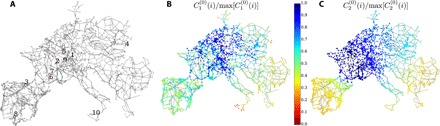
Synchronous high-voltage power grid of continental Europe. (**A**) Topology of the European electric power grid (see Materials and Methods and the Supplementary Materials) and location of the 10 test nodes listed in Table 1. Normalized generalized resistance centralities $C_{1}^{\left(0\right)}(i)$ (**B**) and $C_{2}^{\left(0\right)}(i)$ (**C**) for the network Laplacian matrix of the European electric power grid.

The generalized resistance centralities and Kirchhoff indices appearing in Eq. 6 depend on the operational state via the weighted Laplacian $L(\theta^{\left(0\right)})$. For a narrow distribution of natural frequencies *P_i_* ≪ ∑*_j_b_ij_*, ∀*i*, angle differences between coupled nodes remain small, and the weighted Laplacian is close to the network Laplacian, $L(\theta^{\left(0\right)})≃L^{\left(0\right)}$. The resistance centralities $C_{1}^{\left(0\right)}$ and $C_{2}^{\left(0\right)}$ for the network Laplacian of the European electric power grid (see Materials and Methods and the Supplementary Materials) are shown in Fig. 2. For both centralities, the less central nodes are dominantly located in the Balkans and Spain. In addition, for $C_{1}^{\left(0\right)}$, nodes in Denmark and Sicily are also among the most peripheral. The general pattern of these most peripheral nodes looks very similar to the pattern of most sensitive nodes numerically found in (*47*) and includes particularly many, but not all dead ends, which have been numerically found to undermine grid stability (*48*).

The asymptotic results of Eq. 6, together with the numerical results of Fig. 1, make a strong point that nodal sensitivity to fast or slowly decorrelating noise disturbances can be predicted by generalized resistance centralities. One may wonder at this point how generalized resistance centralities differ in that prediction from other, more common centralities such as geodesic centrality, nodal degree, or PageRank. Table 1 compares these centralities to each other and to the performance measures corresponding to slowly decorrelating noisy disturbances acting on the 10 nodes shown in Fig. 2A. As expected from Eq. 6, 𝒫_1_ and 𝒫_2_ are almost perfectly correlated with the inverse resistance centralities $C_{2}^{-1}$ and $C_{1}^{-1}$, respectively, but with no other centrality metrics. For the full set of nodes of the Europen electric power grid, we found Pearson correlation coefficients $\rho (P_{1},C_{2}^{-1})=0.997$ and $\rho (P_{2},C_{1}^{-1})=0.975$ fully corroborating the prediction of Eq. 6.

**Table 1:** Centrality metrics and performance measures ${𝒫}_{1,2}$ for the European electric power grid (see Materials and Methods and the Supplementary Materials) with noisy disturbances with large correlation time τ_0_ applied on the nodes shown in Fig. 2A. The performance measures 𝒫_1_ and 𝒫_2_ are almost perfectly correlated with the inverse resistance centralities *C*_2_^−1^ and *C*_1_^−1^, respectively, but neither with the geodesic centrality, nor the degree, nor PageRank.

**Node no.**	***C*_geo_**	**Degree**	**PageRank**	***C*_1_**	***C*_2_**	**${𝒫}_{1}^{\text{num}}$**	**${𝒫}_{2}^{\text{num}}$ (γ^2^)**
1	7.84	4	2782	31.86	5.18	0.047	0.035
2	6.8	1	199	22.45	5.68	0.021	0.118
3	5.56	10	3802	22.45	2.33	0.32	0.116
4	4.79	3	362	21.74	3.79	0.126	0.127
5	7.08	1	1217	21.74	5.34	0.026	0.125
6	4.38	6	3091	21.69	5.65	0.023	0.129
7	5.11	2	445	19.4	5.89	0.016	0.164
8	4.15	6	3648	19.38	1.83	0.453	0.172
9	5.06	1	8	10.2	5.2	0.047	0.449
10	2.72	4	3124	7.49	2.17	0.335	0.64

## DISCUSSION

Once a one-to-one relation between the generalized resistance centralities *C*_1_(*k*) and *C*_2_(*k*) of the disturbed node *k* and the magnitude of the induced transient response is established, ranking of nodes from most to least critical is tantamount to ranking them from smallest to largest *C*_1_ or *C*_2_. From Eq. 6, which of these two centralities is relevant depends on whether one is interested (i) in the transient response under fast or slowly decorrelating noise or (ii) in investigating transient behaviors for angles (using the performance measure 𝒫_1_) or frequencies (𝒫_2_). While this gives a priori four different rankings, Eq. 6 leads to only two rankings, based on either $C_{1}^{-1}$ or $C_{2}^{-1}$, which can be obtained through the performance measure 𝒫_1_ only, in asymptotic limit of either very fast (shortest time scale τ_0_) or very slowly (largest τ_0_) decorrelating noise. From here on, we therefore focus on the angle performance measure 𝒫_1_ of Eq. 3a and consider the two asymptotic limits in Eq. 6a.

**Fig. 3:**
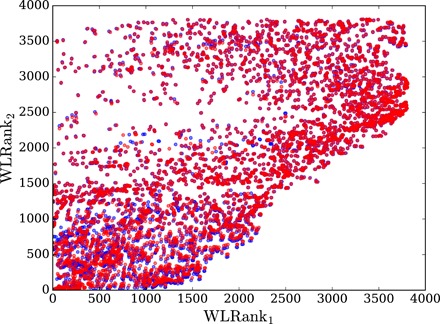
Comparison of the two nodal rankings WLRank_1_ and WLRank_2_ obtained from the generalized resistance centralities *C*_1_ and *C*_2_, respectively, for the 3809 nodes of the European electric power grid sketched in Fig. 2A (see Materials and Methods and the Supplementary Materials). Blue dots correspond to a moderate load during a standard winter weekday, and red dots correspond to a significantly heavier load corresponding to the exceptional November 2016 situation with a rather large consumption and 20 French nuclear reactors shut down.

We therefore define WLRank_1_ and WLRank_2_ (*49*) as two rankings, which order nodes from smallest to largest *C*_1_ and *C*_2_, respectively. Smallest WLRank_1,2_ therefore identify the most vulnerable nodes in a given network. Figure 3 shows that they differ very significantly. In particular, a number of nodes are among the most critical according to WLRank_1_ but not to WLRank_2_ and vice versa. This discrepancy means that nodes are not central in an absolute sense, instead, their centrality and hence how critical they are depend on details of the disturbance—in the present case, the correlation time τ_0_—and the performance measure of interest. One should therefore choose to use one or the other centrality measure, according to the network sensitivity one wants to check.

**Fig. 4:**
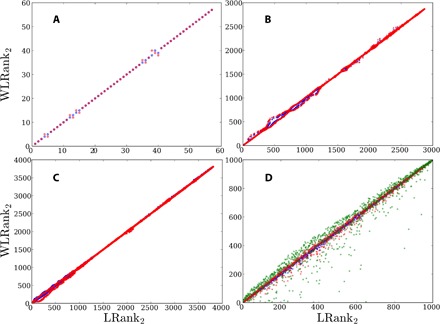
Comparison between LRank and WLRank corresponding to ${𝒫}_{1}$ for noisy disturbances with large correlation time τ_0_. (**A** to **C**) Electric power grid models for normally (blue) and more heavily loaded (red) operating states governed by Eq. 1. (A) IEEE 57 bus test case where the more loaded case has injections six times larger than the moderately loaded, tabulated case (*52*). (B) MATPOWER Pegase 2869 test where the more loaded case has injections 30% larger than the moderately loaded, tabulated case (*53*). (C) European electric power grid model sketched in Fig. 2A (see Materials and Methods and the Supplementary Materials) where the moderately loaded case corresponds to a standard winter weekday and the more heavily loaded case to the November 2016 situation with 20 French nuclear reactors offline. For both cases, the operational state is obtained from an optimal power flow including physical, technological, and economic constraints (see Materials and Methods and the Supplementary Materials). (**D**) Inertialess coupled oscillators governed by Eq. 1 with *m_i_* = 0, **∀***i*, on a random network with 1000 nodes obtained by rewiring a cyclic graph with constant nearest and next-to-nearest neighbor coupling with a probability of 0.5 (see Materials and Methods and the Supplementary Materials) (*50*). Natural frequencies are randomly distributed as *P_i_* ∈ [ − 1.8,1.63] (blue), *P_i_* ∈ [ − 2.16,1.95] (red), and *P_i_* ∈ [ − 2.7,2.45] (green), corresponding to maximal angle differences max(Δθ) = 31^o^, 70^o^, and 106^o^, respectively.

The resistance centralities in Eq. 6 correspond to the network defined by the weighted Laplacian $L(\theta^{\left(0\right)})$ defined in Eq. 2. They therefore depend on the unperturbed, operating state **θ**^(0)^, and consequently, WLRank depends not only on the network topology but also, as expected, on the natural frequencies and the coupling between the nodal degrees of freedom. As mentioned above, in the strong coupling limit, angle differences between coupled nodes remain small and $L(\theta^{\left(0\right)})≃L^{\left(0\right)}$. In that limit, one therefore expects nodal ranking to be given by resistance distances corresponding to the network Laplacian $L^{\left(0\right)}$. How long this remains true is of central interest, and to answer this question, we define further rankings LRank_1,2_ as the rankings using resistance centralities $C_{1,2}^{\left(0\right)}$ obtained from the network Laplacian $L^{\left(0\right)}$. As long as angle differences between network-coupled nodes are not too large, the ranking LRank based on the network Laplacian matrix is almost the same as the true ranking WLRank based on the weighted Laplacian. This is shown in Fig. 4 for three electric power grid models and one random network of coupled oscillators. For the electric power grid models, injections/natural frequencies are limited by the standard operational constraint that the thermal limit of each power line is, at most, only weakly exceeded. This corresponds approximately to a maximal angle difference of max(Δθ) ≃ 30° between any pair of coupled nodes. Accordingly, we find that even in relatively strongly loaded power grids (corresponding, for instance, to the exceptional situation of the fall of 2016 when 20 French nuclear reactors were simultaneously offline; see red points in Fig. 4C), there is not much of a difference between LRank and WLRank. The two rankings start to differ from one another only when at least some natural frequencies become comparable with the corresponding nodal index, *P_i_* ≲ ∑*_j_b_ij_*, and angle differences become very large. This case has been investigated for an inertialess coupled oscillator system on a random rewired network with constant couplings (see Materials and Methods and the Supplementary Materials) (*50*). It is shown in green in Fig. 4D and corresponds to max(Δθ) = 106°.

**Fig. 5:**
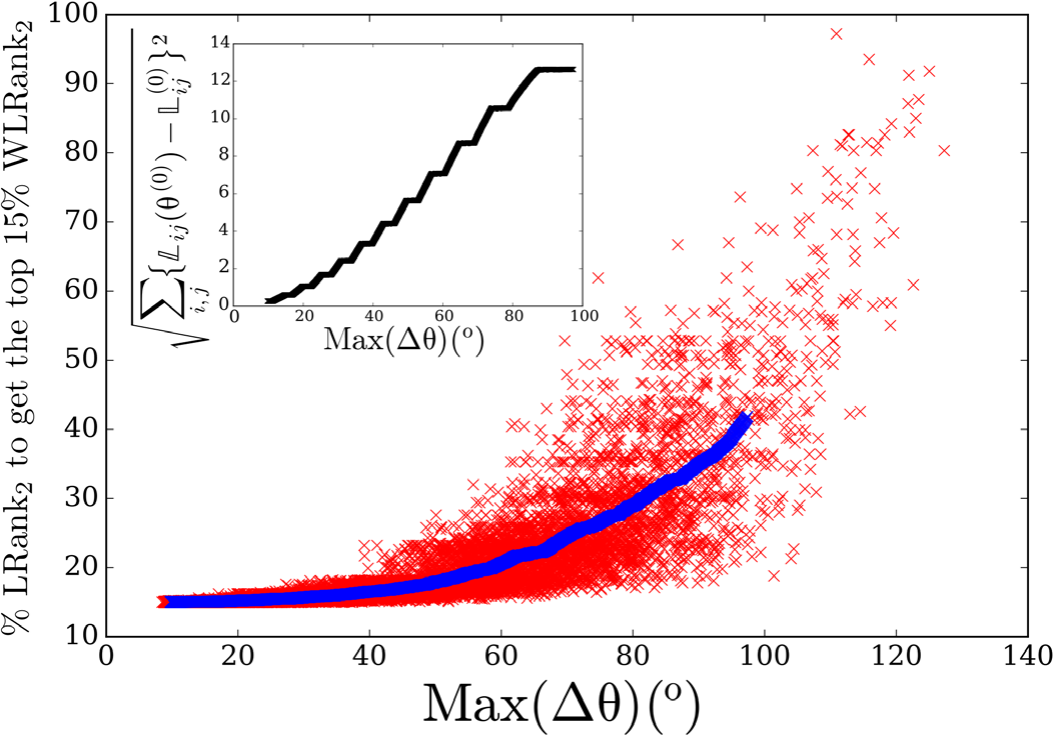
Percentage of the nodes with highest LRank_2_ necessary to give the top 15% ranked nodes with WLRank_2_ for a random network of inertialess coupled oscillators with 1000 nodes obtained by rewiring with a probability of 0.5 of a cyclic network with constant nearest and next-to-nearest neighbor coupling (see Materials and Methods and the Supplementary Materials) (*50*). Each of the 12,000 red crosses corresponds to one of 1000 random natural frequency vector **P**^(0)^ with components randomly distributed in (−0.5,0.5) and summing to zero, multiplied by a prefactor β = 0.4,0.6,…,2.4,2.6. The blue crosses correspond to running averages more than 500 red crosses with consecutive values of max(Δθ). Inset: Running averages of the Frobenius distance between the matrices $L(\theta^{\left(0\right)})$ and $L^{\left(0\right)}$. The steps in the curve reflect discrete increments of β.

In Fig. 5, we investigate more closely when the approximate ranking LRank starts to differ from the true ranking WLRank. To that end, we used the randomly rewired model of inertialess coupled oscillators of Fig. 4D and calculated the percentage of nodes with highest LRank necessary to give the top 15% ranked nodes with WLRank_2_. The results are plotted as a function of the maximal angle difference between directly coupled nodes. Each of the 12,000 red crosses in Fig. 5 corresponds to one of 1000 natural frequency vectors **P**^(0)^, with components randomly distributed in (−0.5,0.5) and summing to zero, multiplied by a prefactor β = 0.4,0.6,…2.4,2.6. The blue crosses correspond to running averages more than 500 red crosses with consecutive values of max(Δθ). One sees that, up to almost max(Δθ) ≃ 40°, the set of the 18% of nodes with highest LRank_2_ always includes the top 15% ranked nodes with WLRank_2_. Similar results for obtaining the top 10 and 20% ranked nodes with WLRank_2_ and for rankings using *C*_1_ instead of *C*_2_ are shown in Materials and Methods and the Supplementary Materials.

It is quite unexpected that nodal ranking remains almost the same up to angle differences of about 40°, since coupling nonlinearities are already well developed there. This is illustrated in the inset of Fig. 5, which plots the Frobenius distance $\sqrt{\sum_{\mathrm{ij}}{\left(L_{\mathrm{ij}}(\theta^{\left(0\right)})-L_{\mathrm{ij}}^{\left(0\right)}\right)}^{2}}$ between the network Laplacian $L^{\left(0\right)}$ and the weighted Laplacian $L(\theta^{\left(0\right)})$. When max(Δθ) ≃ 40°, the Frobenius distance has already reached about 27% of its maximal observed value, indicating that coupling nonlinearities are already strong. Yet, obtaining a desired set of the *n*_s_ most critical nodes for any configuration with max(Δθ) ≲ 40°, including cases with nonegligible nonlinearities, is achieved with a single matrix inversion of the network Laplacian $L^{\left(0\right)}$, while considering a slightly extended set of *n*_s_ + δ*n*_s_ nodes with highest LRank, δ*n*_s_/*n*_s_ ≪ 1. This is a moderate price to pay, compared to the price of calculating WLRank for each configuration, which each time requires inverting the weighted Laplacian matrix $L(\theta^{\left(0\right)})$. That latter procedure would be too time consuming for real-time assessment of large networks.

**Fig. 6:**
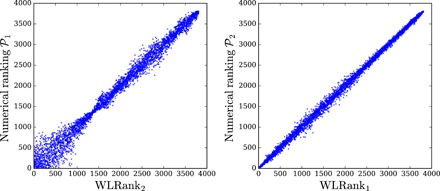
Comparison between WLRank and numerical ranking for systems with inhomogeneous inertia and damping parameters. (**Left**) Numerically obtained ranking based on the performance measure 𝒫_1_ plotted against the ranking WLRank_2_ based on the centrality *C*_2_ and (**right**) numerically obtained ranking based on the performance measure 𝒫_2_ plotted against the ranking WLRank_1_ based on the centrality *C*_1_. Each point is an average *over* more than 40 different noisy disturbances on a single node of the European electric power grid sketched in Fig. 2A, with independently fluctuating damping and inertia coefficients, *d_i_* = *d*_0_ + δ*d_i_* and *m_i_* = *m*_0_ + δ*m_i_* with δ*m_i_*/*m*_0_, δ*d_i_*/*d*_0_ ∈ [−0.4,0.4] and γ = *d*_0_/*m*_0_ = 0.4 s^−1^. The noise correlation time is given by γτ_0_ = 4.

So far, we have assumed constant inertia and damping parameters, which led us to the analytical expressions given in Eq. 6 for the performance measures. Analytical results can further be obtained for inertialess systems with *m_i_* = 0 as well as in the case of homogeneous damping to inertia ratio, *d_i_*/*m_i_* ≡ γ. In this latter case, the ranking is again given by a resistance centrality, but this time, related to the inertia-weighted matrix $M^{-1/2}LM^{-1/2}$ with **M** being the diagonal matrix whose *i*th diagonal entry is given by *m_i_* (see Materials and Methods and the Supplementary Materials) but not in the case of independently varying *m_i_* and *d_i_*. We therefore lastly address this more general case using a purely numerical approach. This question is especially important for electric power grids where only nodes connected to rotating machines (such as conventional power plants) have inertia and consumer nodes have significantly smaller damping parameters (*38*). Time scales in electric power grids have typical values *m_i_*/*d_i_* ∈ [1,3]s and *d_i_*/λ*_α_* ≲ 1s, and accordingly, we focus on the regime of large noise correlation time τ_0_ ≫ *m_i_*/*d_i_*, *d_i_*/λ_α_, which is appropriate for persisting power fluctuations such as those arising from renewable energy sources. Figure 6 shows results corresponding to inertia and damping parameters fluctuating randomly from node to node by up to 40%. The ranking obtained from a full numerical calculation is compared to the ranking obtained from a direct calculation of the centrality of the weighted Laplacian $L_{\mathrm{ij}}(\theta^{\left(0\right)})$, corresponding to the long correlation time asymptotic limit of Eq. 6. One sees that the centrality-based ranking is close to the true, numerically obtained ranking, even in this case of strongly fluctuating inertia and damping parameters. This extends the validity of Eq. 6 for large τ_0_ in a much wider range of parameters than their derivation would suggest.

## CONCLUSION

We have formulated a key player problem in deterministic, network-coupled dynamical systems. The formulation is based on the dynamical response to a nodal additive disturbance of the initial problem, and the most critical nodes—the key players—are defined as those where the response to the disturbance is largest. While this manuscript focused on (i) noisy Ornstein-Uhlenbeck disturbances, (ii) network-coupled systems on undirected graphs, particularly with symmetric couplings *b_ij_* = *b_ji_* in Eq. 1, and (iii) performance measures of the transient response that are quadratic forms in the system’s degrees of freedom, the method is not restricted to these cases. First, it can be used to deal with different disturbances, and in Materials and Methods and the Supplementary Materials, we calculate ${𝒫}_{1,2}$ for a box disturbance δ*P_i_*(*t*) = δ*_ik_*δ*P*_0_Θ(*t*)Θ(τ_0_ − *t*) with the Heaviside function Θ(*t*). This disturbance gives the same ranking as the Ornstein-Uhlenbeck noise disturbance considered above. Second, asymmetric couplings occurring, e.g., in directed graphs (*51*), in Kuramoto models with frustration (*19*), or in electric power grids with Ohmic dissipation (*16*) can also be considered. In this case, the internodal coupling is given by asymmetric real matrices instead of symmetric Laplacian matrices. However, the definition of the resistance distance (Eq. 4) remains valid even if L is replaced by an asymmetric matrix A, in that it still gives Ω*_ii_* = 0, Ω*_ij_* ≥ 0, and Ω*_ij_* ≤ Ω*_ik_* + Ω*_ki_*, ∀*i*, *j*, *k* as long as the synchronous fixed point considered remains stable. Third, nonquadratic performance measures can, in principle, be considered within the spectral decomposition used in this article. One may think of average frequency nadir and rate of change of frequency, which are linear performance measures (*30*, *31*). It is, at present, unclear whether these quantities can be analytically related to the location of disturbances via resistance or other centralities.

We gave an elegant answer to this key player problem: Ranking nodes from most to least critical is tantamount to ranking nodes from least to most central in the sense of resistance centralities. Depending on how the problem is formulated—mostly on details of the disturbance and on how the magnitude of the transient response is measured—different centralities have to be considered, giving different rankings. The key player problem in deterministic systems is therefore not uniquely defined, and its formulation must be tailored to reflect the most relevant dynamical properties one wants to evaluate. Averaged rankings, reflecting several such properties, simultaneously could also be considered. Last, we found numerically that resistance centralities are still accurate to identify the most critical nodes even when nodal dynamical parameters (damping and inertia) are not homogeneous.

The results shown in Fig. 6 are rather unexpected, and further inspection of our analytical results (Eq. 6 and eq. S14B) suggests that an inertia dependence could emerge in the opposite limit of short correlation time τ_0_ ≪ *m_i_*/*d_i_*, *d_i_*/λ_α_. This point deserves further investigations. It would be furthermore interesting to extend our investigations to cases of distributions of inertia and damping parameters corresponding to realistic electric power grids. Work along those lines is in progress.

## MATERIALS AND METHODS

Four different networks were considered. Data for implementing the IEEE 57 bus test case and the MATPOWER Pegase 2869 test case have been obtained from (*52*, *53*). The random networks were obtained through the rewiring procedure of initially regular networks discussed in (*50*). We constructed the European power grid model from publicly available data on power plants and bus locations. More details on the procedure were given in the Supplementary Materials and in (*54*).

Several operational states of the European power grid were obtained from an optimal power flow. The latter minimizes a cost function including production costs for all available power plants, under constraints that power flows do not exceed the thermal line capacity of each power line and that productions do not exceed rated powers for each power plant.

For each network considered, Eq. 1 was numerically integrated using a fourth-order Runge-Kutta algorithm. Values for the performance measures were then calculated by numerical integration of the obtained time-dependent voltage angle and frequencies. Resistance distances, centralities, and Kirchhoff indices were calculated through a direct inversion of the Laplacian matrix.

## Supplementary Material

http://advances.sciencemag.org/cgi/content/full/5/11/eaaw8359/DC1

Download PDF

The key player problem in complex oscillator networks and electric power grids: Resistance centralities identify local vulnerabilities

## SUPPLEMENTARY MATERIALS

Supplementary material for this article is available at http://advances.sciencemag.org/cgi/content/full/5/11/eaaw8359/DC1 Section S1. Calculation of the performance measures Section S2. Resistance distances, centralities, and Kirchhoff indices Section S3. Numerical models Section S4. Numerical comparison of LRank with WLRank. Fig. S1. Comparison between theoretical predictions and numerical results for both performance measures 𝒫_1_ and 𝒫_2_. Fig. S2. Comparison of the performance measures 𝒫_1_, 𝒫_2_ obtained numerically and in eq. S14. Fig. S3. Percentage of the nodes with highest LRank necessary to include the nodes with 10% and 20% highest WLRank. References (*55*, *56*)
