# Supplementary material for: The key player problem in complex oscillator networks and electric power grids: Resistance centralities identify local vulnerabilities
Source: Sci Adv. 2019 Nov 22;5(11):eaaw8359. doi: 10.1126/sciadv.aaw8359 (PMC6874484; doi:10.1126/sciadv.aaw8359)
Supplement: http://advances.sciencemag.org/cgi/content/full/5/11/eaaw8359/DC1 [file supp_5_11_eaaw8359__index.html]

Science Advances | Science AdvancesAAASSearchScience AdvancesMenu

## Supplementary Materials

**This PDF file includes:**

- Section S1. Calculation of the performance measures
- Section S2. Resistance distances, centralities, and Kirchhoff indices
- Section S3. Numerical models
- Section S4. Numerical comparison of LRank with WLRank.
- Fig. S1. Comparison between theoretical predictions and numerical results for both performance measures *P*1 and *P*2.
- Fig. S2. Comparison of the performance measures *P*1, *P*2 obtained numerically and in eq. S14.
- Fig. S3. Percentage of the nodes with highest LRank necessary to include the nodes with 10% and 20% highest WLRank.
- References (*55*, *56*)

Download PDF

**Files in this Data Supplement:**

- Adobe PDF - aaw8359\_SM.pdf
