# Supplementary material for: The key player problem in complex oscillator networks and electric power grids: Resistance centralities identify local vulnerabilities
Source: Sci Adv. 2019 Nov 22;5(11):eaaw8359. doi: 10.1126/sciadv.aaw8359 (PMC6874484; doi:10.1126/sciadv.aaw8359)
Supplement: Download PDF [file aaw8359_SM.pdf]

Science Advances
AAAS

advances.sciencemag.org/cgi/content/full/5/11/eaaw8359/DC1

Supplementary Materials for

The key player problem in complex oscillator networks and electric power grids: Resistance centralities identify local vulnerabilities

M. Tyloo, L. Pagnier, P. Jacquod*

*Corresponding author. Email: philippe.jacquod@hevs.ch

Published 22 November 2019, *Sci. Adv.* **5**, eaaw8359 (2019)
DOI: 10.1126/sciadv.aaw8359

This PDF file includes:

Section S1. Calculation of the performance measures
Section S2. Resistance distances, centralities, and Kirchhoff indices
Section S3. Numerical models
Section S4. Numerical comparison of LRank with WLRank.
Fig. S1. Comparison between theoretical predictions and numerical results for both performance measures $\mathcal{P}_1$ and $\mathcal{P}_2$.
Fig. S2. Comparison of the performance measures $\mathcal{P}_1$, $\mathcal{P}_2$ obtained numerically and in eq. S14.
Fig. S3. Percentage of the nodes with highest LRank necessary to include the nodes with 10% and 20% highest WLRank.
References (*55*, *56*)

Section S1. Calculation of the performance measures

We give some details of the calculation of the performance measures, Eqs. (3) in the main text. These calculations generalize to second-order swing equations the results obtained for the first-order Kuramoto model in Ref. [40]. Starting from Eq. (1) in the main text, we consider a stable fixed-point solution $\boldsymbol{\theta}^{(0)} = (\theta_1^{(0)}, \ldots, \theta_n^{(0)})$ with unperturbed natural frequencies $\boldsymbol{P}^{(0)}$. We subject this state to a time-dependent disturbance $\boldsymbol{P}(t) = \boldsymbol{P}^{(0)} + \delta\boldsymbol{P}(t)$, which makes angles become time-dependent, $\boldsymbol{\theta}(t) = \boldsymbol{\theta}^{(0)} + \delta\boldsymbol{\theta}(t)$. Linearizing the dynamics defined by Eq. (1) of the main text about $\boldsymbol{\theta}^{(0)}$ and under the assumption that $d_i/m_i = \gamma$, $\forall i$, one obtains

$$\delta\ddot{\bar{\boldsymbol{\theta}}} + \gamma\delta\dot{\bar{\boldsymbol{\theta}}} = \boldsymbol{M}^{-1/2}\delta\boldsymbol{P} - \boldsymbol{M}^{-1/2}\mathbb{L}(\boldsymbol{\theta}^{(0)})\boldsymbol{M}^{-1/2}\,\delta\bar{\boldsymbol{\theta}} \tag{S1}$$

where we introduced matrices with elements $D_{ij} = \delta_{ij}\,d_i = \gamma M_{ij}$ and new angle coordinates $\delta\bar{\boldsymbol{\theta}} = \boldsymbol{M}^{1/2}\delta\boldsymbol{\theta}$. The weighted Laplacian matrix $\mathbb{L}(\boldsymbol{\theta}^{(0)})$ is defined as

$$\mathbb{L}_{ij} = \begin{cases} -b_{ij}\cos(\theta_i^{(0)} - \theta_j^{(0)})\,, & i \neq j\,, \\ \sum_k b_{ik}\cos(\theta_i^{(0)} - \theta_k^{(0)})\,, & i = j\,. \end{cases} \tag{S2}$$

This Laplacian is minus the stability matrix of the linearized dynamics about a stable synchronous state. It is therefore positive semidefinite, with its largest eigenvalue $\lambda_1 = 0$ corresponding to a constant eigenvector $\mathbf{u}_1 = (1, 1, 1, ...1)/\sqrt{n}$, and $\lambda_\alpha > 0$, $\alpha = 2, 3, ...n$. We define the matrix $\mathbb{L}^M = \boldsymbol{M}^{-1/2}\mathbb{L}\boldsymbol{M}^{-1/2}$ with eigenvectors $\mathbf{u}_\alpha^M$ and eigenvalues λ_α^M, for $\alpha = 1, 2, ...n$. To calculate the response of the system to $\delta\boldsymbol{P}(t)$, we expand angle deviations over the eigenstates $\mathbf{u}_\alpha^M$ of $\mathbb{L}^M$, $\delta\bar{\boldsymbol{\theta}}(t) = \sum_\alpha c_\alpha(t)\,\mathbf{u}_\alpha^M$. Eq. (S1) becomes

$$\ddot{c}_\alpha(t) + \gamma\dot{c}_\alpha(t) = \boldsymbol{M}^{-1/2}\delta\boldsymbol{P}(t)\cdot\mathbf{u}_\alpha^M - \lambda_\alpha^M c_\alpha(t) \tag{S3}$$

The disturbance starts at $t = 0$ and therefore $\delta\bar{\boldsymbol{\theta}}(0) = 0$ and $\delta\dot{\bar{\boldsymbol{\theta}}}(0) = 0$. Performing a Laplace transform on Eq. (S3), one gets

$$s^2 c_\alpha(s) + \gamma\, s\, c_\alpha(s) = \lambda_\alpha^M c_\alpha(s) + (\boldsymbol{M}^{-1/2}\delta\boldsymbol{P}\cdot\mathbf{u}_\alpha^M)(s) \tag{S4}$$

where $c_\alpha(s) = \int_0^t e^{-st'}c_\alpha(t')dt'$ and $(\boldsymbol{M}^{-1/2}\delta\boldsymbol{P}\cdot\mathbf{u}_\alpha^M)(s) = \int_0^t e^{-st'}\boldsymbol{M}^{-1/2}\delta\boldsymbol{P}(t')\cdot\mathbf{u}_\alpha^M\,dt'$. Finally one obtains the Laplace transformed expansion coefficients of the angles over the eigenbasis of $\mathbf{u}_\alpha^M$ of $\mathbb{L}^M$

$$c_\alpha(s) = (\boldsymbol{M}^{-1/2}\delta\boldsymbol{P}\cdot\mathbf{u}_\alpha^M)(s)\Big/\left(s - \frac{-\gamma + \Gamma_\alpha}{2}\right)\left(s + \frac{\gamma + \Gamma_\alpha}{2}\right) \tag{S5}$$

with $\Gamma_\alpha = \sqrt{\gamma^2 - 4\lambda_\alpha^M}$. Applying an inverse Laplace transform leads to

$$c_\alpha(t) = e^{\frac{-\gamma-\Gamma_\alpha}{2}t}\int_0^t e^{\Gamma_\alpha t'}\int_0^{t'} \boldsymbol{M}^{-1/2}\delta\boldsymbol{P}(t'')\cdot \mathbf{u}_\alpha^M e^{\frac{\gamma-\Gamma_\alpha}{2}t''}dt''dt' \tag{S6}$$

The time-dependence of angle and frequency degrees of freedom is then given by

$$\delta\boldsymbol{\theta}(t) = \boldsymbol{M}^{-1/2}\delta\bar{\boldsymbol{\theta}}(t) = \sum_\alpha c_\alpha(t)\boldsymbol{M}^{-1/2}\mathbf{u}_\alpha^M \tag{S7}$$

$$\delta\dot{\boldsymbol{\theta}}(t) = \boldsymbol{M}^{-1/2}\delta\dot{\bar{\boldsymbol{\theta}}}(t) = \sum_\alpha \dot{c}_\alpha(t)\boldsymbol{M}^{-1/2}\mathbf{u}_\alpha^M \tag{S8}$$

The variances $p_1(t)$ and $p_2(t)$ of the angle and frequency deviations read

$$p_1(t) = \delta\boldsymbol{\theta}^2(t) = \sum_{\alpha,\beta} c_\alpha(t)c_\beta(t)\mathbf{u}_\beta^{M\top}\boldsymbol{M}^{-1}\mathbf{u}_\alpha^M \tag{S9}$$

$$p_2(t) = \delta\dot{\boldsymbol{\theta}}^2(t) = \sum_{\alpha,\beta} \dot{c}_\alpha(t)\dot{c}_\beta(t)\mathbf{u}_\beta^{M\top}\boldsymbol{M}^{-1}\mathbf{u}_\alpha^M \tag{S10}$$

When $d_i = d = \gamma m_i\ \forall i$, both matrices $\mathbb{L}$ and $\mathbb{L}^M$ have the same eigenvectors and $\lambda_\alpha^M = \lambda_\alpha/m$. Below we consider noisy disturbances sequentially for the homogeneous case, $m_i = m$, $d_i = d$, inertialess case, $m_i = 0$ and constant ratio case, $d_i/m_i = \gamma$

A. Correlated Noisy disturbances

1. *Homogeneous Case*

We assume homogeneous inertia and damping factor, respectively $m_i = m$ and $d_i = d$, for the next calculations. In the case of stochastic disturbances that persist in time, we average the p_i's as follows

$$\mathcal{P}_i = \lim_{T\to\infty} T^{-1}\int_0^T \overline{p_i(t)}dt\,,\ i = 1,2 \tag{S11}$$

where $\overline{p_i(t)}$ indicates an average taken over the ensemble defined by e.g. the moments of the stochastic disturbance. We consider Ornstein-Uhlenbeck correlated noise on a single node, k, with zero mean $\overline{\delta P_k(t)} = 0$ and second moment $\overline{\delta P_i(t_1)\delta P_j(t_2)} = \delta_{ik}\delta_{jk}\,\delta P_0^2\exp[-|t_1 - t_2|/\tau_0]$,

correlated over a typical time scale τ_0. We have

$$\mathcal{P}_1 = \lim_{T\to\infty} T^{-1} \sum_{\alpha\geq 2} \int_0^T \overline{c_\alpha^2(t)} dt \tag{S12}$$

$$= \lim_{T\to\infty} T^{-1} \sum_{\alpha\geq 2} \int_0^T e^{-(\gamma+\Gamma_\alpha)t} \int_0^t \int_0^t e^{\Gamma_\alpha(t_1'+t_2')} \times \tag{S13}$$

$$\int_0^{t_1'} \int_0^{t_2'} \sum_{i,j} \frac{u_{\alpha,i} u_{\alpha,j}}{m} \overline{\delta P_i(t_1'') \delta P_j(t_2'')}\, e^{\frac{\gamma-\Gamma_\alpha}{2}(t_1''+t_2'')} \, dt dt_1' dt_2' dt_1'' dt_2''$$

For homogeneous damping and inertia one has $\Gamma_\alpha = \sqrt{\gamma^2 - 4\lambda_\alpha/m}$. The integrals can be performed straightforwardly and one obtains

$$\mathcal{P}_1 = \delta P_0^2 \sum_{\alpha\geq 2} \frac{u_{\alpha,k}^2 (\tau_0 + m/d)}{\lambda_\alpha(\lambda_\alpha \tau_0 + d + m\tau_0^{-1})} \tag{S14a}$$

$$\mathcal{P}_2 = \delta P_0^2 \sum_{\alpha\geq 2} \frac{u_{\alpha,k}^2}{d(\lambda_\alpha \tau_0 + d + m\tau_0^{-1})} \tag{S14b}$$

Taking the two limits $\lambda_\alpha \tau_0 \gg d$, $\lambda_\alpha \tau_0^2 \gg m$ and $\lambda_\alpha \tau_0 \ll d$, $\lambda_\alpha \tau_0^2 \ll m$, Eqs. (6a,b) of the main text are then easily obtained.

2. *Inertialess case*

The performance measures for Kuramoto oscillators are obtained from Eqs. (S14) with $m = 0$ [40]

$$\mathcal{P}_1 = \delta P_0^2 \sum_{\alpha\geq 2} \frac{u_{\alpha,k}^2 \tau_0}{\lambda_\alpha(\lambda_\alpha \tau_0 + d)} \tag{S15a}$$

$$\mathcal{P}_2 = \delta P_0^2 \sum_{\alpha\geq 2} \frac{u_{\alpha,k}^2}{d(\lambda_\alpha \tau_0 + d)} \tag{S15b}$$

The asymptotics are then obtained by taking the asymptotic limits of large/small τ_0 only after setting $m = 0$. One obtains

$$\mathcal{P}_1 = \begin{cases} \big(\delta P_0^2 \tau_0\big)/d)\, \big(C_1^{-1}(k) - n^{-2} Kf_1\big) \;\;, \; \lambda_\alpha \tau_0 \ll 1\,, \\ \delta P_0^2 \, \big(C_2^{-1}(k) - n^{-2} Kf_2\big) \;\;, \; \lambda_\alpha \tau_0 \gg d\,, \end{cases} \tag{S16a}$$

$$\mathcal{P}_2 = \begin{cases} \big(\delta P_0^2 \tau_0/d\big)\big(n-1\big)/n \;\;, \; \lambda_\alpha \tau_0 \ll 1\,, \\ \big(\delta P_0^2/d\tau_0\big)\, \big(C_1^{-1}(k) - n^{-2} Kf_1\big) \;\;, \; \lambda_\alpha \tau_0 \gg d\,, \end{cases} \tag{S16b}$$

where we use the generalized resistance centralities $C_{1,2}(i)$ and Kirchhoff indices $Kf_{1,2}$ discussed in Section II below.

3. Constant inertia to damping ratio

The cases of varying m_i and d_i can be further treated analytically, provided the ratio $d_i/m_i = \gamma$ remains constant. The price to pay is to include inertia coefficients in the performance measures and consider

$$\mathcal{P}_1 = \lim_{T\to\infty} T^{-1} \sum_i m_i \int_0^T \overline{|\delta\theta_i(t) - \Delta(t)|^2}\, \mathrm{d}t \tag{S17a}$$

$$\mathcal{P}_2 = \lim_{T\to\infty} T^{-1} \sum_i m_i \int_0^T \overline{|\delta\dot{\theta}_i(t) - \dot{\Delta}(t)|^2}\, \mathrm{d}t \tag{S17b}$$

Note that this is not a fundamental redefinition, since all previously obtained results in the case of constant inertia and damping can be multiplied by $m_i \equiv m$ for comparison with results about to be presented. Performance measures are then obtained in a similar way as for the homogeneous case. They read

$$\mathcal{P}_1 = \frac{\delta P_0^2}{m_k} \sum_{\alpha\geq 2} \frac{{u_{\alpha,k}^M}^2(\tau_0\gamma + 1)}{\gamma\lambda_\alpha^M(\lambda_\alpha^M\tau_0 + \gamma + \tau_0^{-1})} \tag{S18a}$$

$$\mathcal{P}_2 = \frac{\delta P_0^2}{m_k} \sum_{\alpha\geq 2} \frac{{u_{\alpha,k}^M}^2}{\gamma(\lambda_\alpha^M\tau_0 + \gamma + \tau_0^{-1})} \tag{S18b}$$

Here, λ_α^M, $\mathbf{u}_\alpha^M$ are respectively the eigenvalues and eigenvectors of the matrix $\boldsymbol{M}^{-1/2}\mathbb{L}\boldsymbol{M}^{-1/2}$. Similar expressions were obtained for other performance measures such as kinetic energy, primary control effort or line dissipation [41,42,43,46]. In both limits $\tau_0 \ll \gamma^{-1}, {\lambda_\alpha^M}^{-1/2}$ and $\tau_0 \gg \gamma^{-1}, {\lambda_\alpha^M}^{-1/2}$ performance measures $\mathcal{P}_1$, $\mathcal{P}_2$ can be expressed in terms of resistance centralities related to $\boldsymbol{M}^{-1/2}\mathbb{L}\boldsymbol{M}^{-1/2}$ (see Eq. (S26) with $\mathbb{L}' = \boldsymbol{M}^{-1/2}\mathbb{L}\boldsymbol{M}^{-1/2}$) and the inertia m_k of the perturbed node.

B. Box disturbances

The same kind of computation as for the noisy disturbance can be done with a box disturbance acting on node k, i.e. $\delta P_i(t) = \delta_{ik}\, \delta P_0\, \Theta(t)\Theta(\tau_0 - t)$ with the Heaviside step function $\Theta(t) = 0$ for $t < 0$ and $\Theta(t) = 1$ for $t \geq 1$. As the perturbation is limited in time,

we consider the performance measures

$$\mathcal{P}_1^\infty = \sum_i \int_0^\infty |\delta\theta_i - \Delta(t)|^2 dt \tag{S19}$$

$$\mathcal{P}_2^\infty = \sum_i \int_0^\infty |\delta\dot{\theta}_i - \dot{\Delta}(t)|^2 dt \tag{S20}$$

instead of (S11). For uniform inertia and damping one obtains

$$\mathcal{P}_1^\infty = \frac{\delta P_0^2 m}{8\gamma} \sum_{\alpha\geq 2} \frac{u_{\alpha,k}^2}{\Gamma_\alpha \lambda_\alpha^3} \left[2\Gamma_\alpha(4\gamma\tau_0\lambda_\alpha/m - 3\gamma^2 - \Gamma_\alpha^2) + (\gamma+\Gamma_\alpha)^3 e^{-\tau_0\frac{(\gamma-\Gamma_\alpha)}{2}} - (\gamma-\Gamma_\alpha)^3 e^{-\tau_0\frac{(\gamma+\Gamma_\alpha)}{2}}\right],$$

$$\mathcal{P}_2^\infty = \frac{\delta P_0^2}{2d} \sum_{\alpha\geq 2} \frac{u_{\alpha,k}^2}{\Gamma_\alpha \lambda_\alpha} \left[2\Gamma_\alpha - (\gamma+\Gamma_\alpha) e^{-\frac{\tau_0(\gamma-\Gamma_\alpha)}{2}} + (\gamma-\Gamma_\alpha) e^{-\frac{\tau_0(\gamma+\Gamma_\alpha)}{2}}\right]$$

with $\Gamma_\alpha = \sqrt{\gamma^2 - 4\lambda_\alpha/m}$. The two asymptotic limits of large and small τ_0 are given by

$$\mathcal{P}_1^\infty = \begin{cases} \left(\delta P_0^2 \tau_0^2/2d\right)\left(C_1^{-1}(k) - n^{-2}Kf_1\right) & , (\gamma \pm \Gamma_\alpha)\tau_0 \ll 1\,, \\ \delta P_0^2 \tau_0 \left(C_2^{-1}(k) - n^{-2}Kf_2\right) & , (\gamma \pm \Gamma_\alpha)\tau_0 \gg 1 \text{ and } \lambda_\alpha\tau_0/d \gg 1 \end{cases} \tag{S21a}$$

$$\mathcal{P}_2^\infty = \begin{cases} \left(\delta P_0^2 \tau_0^2/2md\right)\left(n-1\right)/n & , (\gamma \pm \Gamma_\alpha)\tau_0 \ll 1\,, \\ \left(\delta P_0^2/d\right)\left(C_1^{-1}(k) - n^{-2}Kf_1\right) & , (\gamma \pm \Gamma_\alpha)\tau_0 \gg 1 \end{cases} \tag{S21b}$$

which are also given by resistance centralities and Kirchhoff indices.

Section S2. Resistance distances, centralities, and Kirchhoff indices

The resistance centralities C_1 and C_2 can be expressed as functions of the distribution of resistance distances Ω_{ij}, between any pairs of nodes (i,j) of the network. The Laplacian matrix $\mathbb{L}$ of the network has one zero eigenvalue associated to the constant eigenvector $u_{1,i} = 1/\sqrt{n}$, its pseudoinverse $\mathbb{L}^\dagger$ is defined by [36]

$$\mathbb{L}\mathbb{L}^\dagger = \mathbb{L}^\dagger\mathbb{L} = \mathbb{1} - \mathbf{u}_1^\top \mathbf{u}_1 \tag{S22}$$

from which the resistance distance between nodes i and j is expressed as

$$\Omega_{ij} = \mathbb{L}_{ii}^\dagger + \mathbb{L}_{jj}^\dagger - \mathbb{L}_{ij}^\dagger - \mathbb{L}_{ji}^\dagger \tag{S23}$$

Using the eigenvectors of $\mathbb{L}$ we can rewrite Eq. (S23) as [40]

$$\Omega_{ij} = \sum_{\alpha\geq 2} \frac{(u_{\alpha,i} - u_{\alpha,j})^2}{\lambda_\alpha} \tag{S24}$$

The resistance distance is a graph metric in the sense that : i) $\Omega_{ii} = 0$, $\forall i$, ii) $\Omega_{ij} \geq 0$, $\forall i, j$, and iii) $\Omega_{ij} + \Omega_{jk} \geq \Omega_{ik}$, $\forall i, j, k$ (triangle inequality) [36]. The Kirchhoff index of a network is obtained from the resistance distances by summing over all pairs of nodes [36]

$$Kf_1 = \sum_{i<j} \Omega_{ij} = n \sum_{\alpha \geq 2} \lambda_\alpha^{-1} \tag{S25}$$

The Kirchhoff index is, up to a normalization factor, the mean resistance distance over the whole graph.

We generalize this definition of the resistance distance for matrices that are powers of the original Laplacian matrix, $\mathbb{L}' = \mathbb{L}^p$ and thus $[\mathbb{L}']^\dagger = \left[\mathbb{L}^p + \mathbf{u}_1^\top \mathbf{u}_1\right]^{-1}$. One has

$$\Omega_{ij}^{(p)} = [\mathbb{L}'^\dagger]_{ii} + [\mathbb{L}'^\dagger]_{jj} - [\mathbb{L}'^\dagger]_{ij} - [\mathbb{L}'^\dagger]_{ji} \tag{S26}$$

The eigenvectors of $\mathbb{L}'$ are the same as those of $\mathbb{L}$. Thus we have

$$\Omega_{ij}^{(p)} = \sum_{\alpha \geq 2} \frac{(u_{\alpha,i} - u_{\alpha,j})^2}{\lambda_\alpha^p} \tag{S27}$$

We still have to check that the generalized resistance distances $\Omega_{ij}^{(p)}$ have the three properties of a graph metric. We remark that $\Omega_{ij}^{(p)}$ corresponds to the resistance distance between nodes i and j in a new graph whose Laplacian is $\mathbb{L}' = \mathbb{L}^p$. Therefore it is sufficient to show that $\mathbb{L}'$ is also a Laplacian matrix. to that end we demonstrate that the product of two Laplacian matrices $\mathcal{A}$ and $\mathcal{B}$ is still a Laplacian matrix. For a Laplacian matrix $\mathcal{A}$ one has (i) $\sum_i \mathcal{A}_{ij} = 0$, (ii) $\mathcal{A}_{ii} = -\sum_{j \neq i} \mathcal{A}_{ij}$. From these generic properties of Laplacian matrices, matrix elements of the product $\mathcal{AB}$ satisfy

$$\sum_j [\mathcal{AB}]_{ij} = \sum_{j,k} \mathcal{A}_{ik} \mathcal{B}_{kj} = 0 \tag{S28}$$

$$\sum_{j \neq i} [\mathcal{AB}]_{ij} = \sum_j [\mathcal{AB}]_{ij} - [\mathcal{AB}]_{ii} = -[\mathcal{AB}]_{ii} \tag{S29}$$

We conclude that the product $\mathcal{AB}$ is also a Laplacian matrix, and therefore, the generalized resistance distances $\Omega_{ij}^{(p)}$ have the three properties of a graph metric. With the generalized resistance distances, we can define generalized Kirchhoff indices [40]

$$Kf_p = \sum_{i<j} \Omega_{ij}^{(p)} = n \sum_{\alpha \geq 2} \lambda_\alpha^{-p} \tag{S30}$$

The relation between the resistive centrality $C_1(i)$ and the resistance distance is obtained from Eqs. (S24) and (S25)

$$C_1(i) = \left[n^{-1}\sum_j \Omega_{ij}\right]^{-1} = \left[\sum_{\alpha\geq 2}\frac{u^2_{\alpha,i}}{\lambda_\alpha} + n^{-2}Kf_1\right]^{-1} \tag{S31}$$

The expression for $C_2(i)$ involves higher moments of the distribution of resistance distances. We obtain

$$C_2(i) = \sum_j \Omega^2_{ij} - n\,C_1^{-2}(i) + 2\sum_j \Omega_{ij}\,C_1^{-1}(j) - 4\,C_1^{-1}(i)\,n^{-1}Kf_1 - 3\sum_j C_1^{-2}(j) + 12n^{-3}Kf_1^2$$

Section S3. Numerical models

We checked our analytical results against numerical ones obtained for four different models which we briefly describe here.

A. European electric power grid

We have constructed a model of the European high voltage electrical grid. It is composed of 3809 consumer and generator nodes connected to one another by 4944 lines. The geographic location of each node and the location of the lines between them has been extracted from the ENTSO-E database [55]. Line capacities b_{ij} between nodes have been normalized proportionally to the inverse of their length as follows

$$b_{ij} = 1/(X_{ij}l_{ij}) \tag{S32}$$

with l_{ij} the length of the line (in kilometers) and X_{ij} its kilometric reactance. We checked our model of the European power grid against (i) available data for a part of the French transmission grid [56] and (ii) exact Swissgrid data for the Swiss transmission grid. Doing so, we found discrepancies below 13%. The operational states (injections and consumptions) of the power grid are obtained via an optimal power flow which constrains the load flows on each line with the thermal limit of the latter and takes into account technical specificities for each power plant [39,53,54]. The two operational states considered in Figs. 3 and 4c of the main text correspond to a typical electric power consumption situation in winter (blue) and a case reproducing the extraordinary situation of November 2016, with a relatively high power

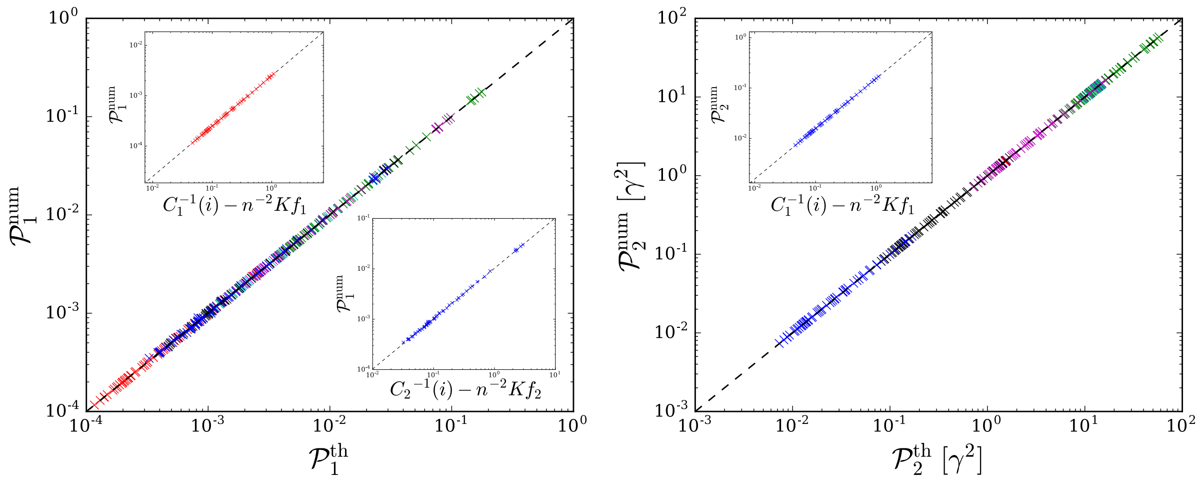

Fig. S1. Comparison between theoretical predictions and numerical results for both performance measures $\mathcal{P}_1$ and $\mathcal{P}_2$. Each point corresponds to a noisy disturbance on a single node of the IEEE 57 bus test case [52] with magnitude $\delta P_0 = 0.1$ and correlation times $\gamma\tau_0 = 4 \cdot 10^{-4}$ (red crosses), $4 \cdot 10^{-3}$ (cyan), $4 \cdot 10^{-2}$ (green), $4 \cdot 10^{-1}$ (purple), 4 (black) and 40 (blue). Time scales are defined by the ratio of damping to inertia coefficients $\gamma = d_i/m_i = 0.4s^{-1}$ which is assumed constant with $d_i = 0.004s$. The insets show $\mathcal{P}_1$ and $\mathcal{P}_2$ as a function of the resistance distance-based graph-theoretic predictions of Eqs. (5) in the main text, valid in both limits of very large and very short noise decorrelation time τ_0. Not shown is the limit of short τ_0 for $\mathcal{P}_2$, which gives a node-independent result.

demand and twenty french nuclear reactors offline (red). For the numerical simulations in Fig. 1 of the main text, we used the first case. Rotating machines are characterized by the inertia constant H_i which is the time over which the rated power of the generator provides a work equivalent to its kinetic energy [16]. For this model, the network Laplacian matrix has a spectrum distributed in the interval $\lambda_\alpha \in [0.0458, 26678.4395]$ (in the per unit system [16]). The typical time scales of the system are $d/\lambda_\alpha < 0.4s$ and $m/d \cong 2.5s$.

B. IEEE 57 bus test case

The IEEE 57 bus test case is a standardly used model of an electric power grid [52]. It is composed of 57 buses including 7 generators and 80 lines. In Fig.4a of the main text, we use the tabulated operational state as well as a state where the tabulated loads are

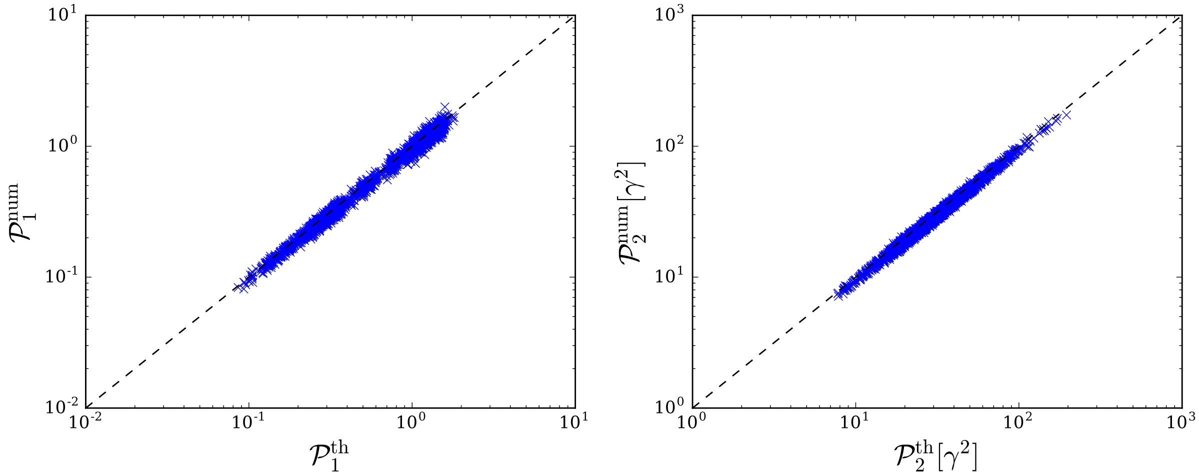

Fig. S2. Comparison of the performance measures $\mathcal{P}_1$, $\mathcal{P}_2$ obtained numerically and in eq. S14. Each point corresponds to a noisy disturbance on a single node of the Pegase 2869 test case [53] with magnitude $\delta P_0 = 0.1$ and correlation time $\gamma\tau_0 = 0.4$ and ratio of damping to inertia $\gamma = 0.4s^{-1}$ with $d_i = 0.007s$.

increased by a factor six [52]. The spectrum of the Laplacian is distributed in the interval $\lambda_\alpha \in [0.2796, 118.6186]$ (in the per unit system [16]).

Fig. S1 shows data similar to Fig. 1 in the main text for the IEEE 57 bus test case. The insets shows the asymptotic limits of very large and very small τ_0, where $\mathcal{P}_{1,2}$ are predicted to be linear functions of the resistance centralities $C_{1,2}$ (see main text).

C. MATPOWER Pegase 2869 Test Case

The MATPOWER test case Pegase 2869 is a model representing a part of the European high voltage transmission grid [53]. It is composed of 2869 buses including 510 generators and 4582 lines. In Fig.4b of the main text, we use the tabulated operational state as well as a state where injections are 30% larger [53].The spectrum of the Laplacian is distributed in the interval $\lambda_\alpha \in [0.03536, 27156.901]$ (in the per unit system [16]). Fig. S2 shows data similar to Fig. 1 in the main text for this model.

D. Random Network

We finally used a random network obtained by random rewiring of edges with probability 0.5 of a single-cycle network with 1000 nodes with nearest and next-to-nearest couplings [50]. Edges have the same weight $b_{ij} = b_0 = 1s^{-1}$. The spectrum of the Laplacian is distributed in the interval $\lambda_\alpha \in [0.39b_0, 10.47b_0]$.

In our numerics, we define a first-order, inertialess Kuramoto model on this random network. Fig.4d of the main text considers various distribution of natural frequencies, including one (green) which is close to instability with angle differences larger than 90°.

Section S4. Numerical comparison of LRank with WLRank

In Fig.5 of the main text, we calculated the percentage of nodes with highest LRank_2 necessary to give the top 15 % ranked nodes with WLRank_2. The conclusions drawn from these data are generic – they are valid for different percentages than 15% and for LRank_1 vs. WLRank_1. This is illustrated in fig. S3, which shows similar results for the percentage of nodes with highest $\mathrm{LRank}_{1,2}$ that include the top 10% and 20% ranked nodes with $\mathrm{WLRank}_{1,2}$.

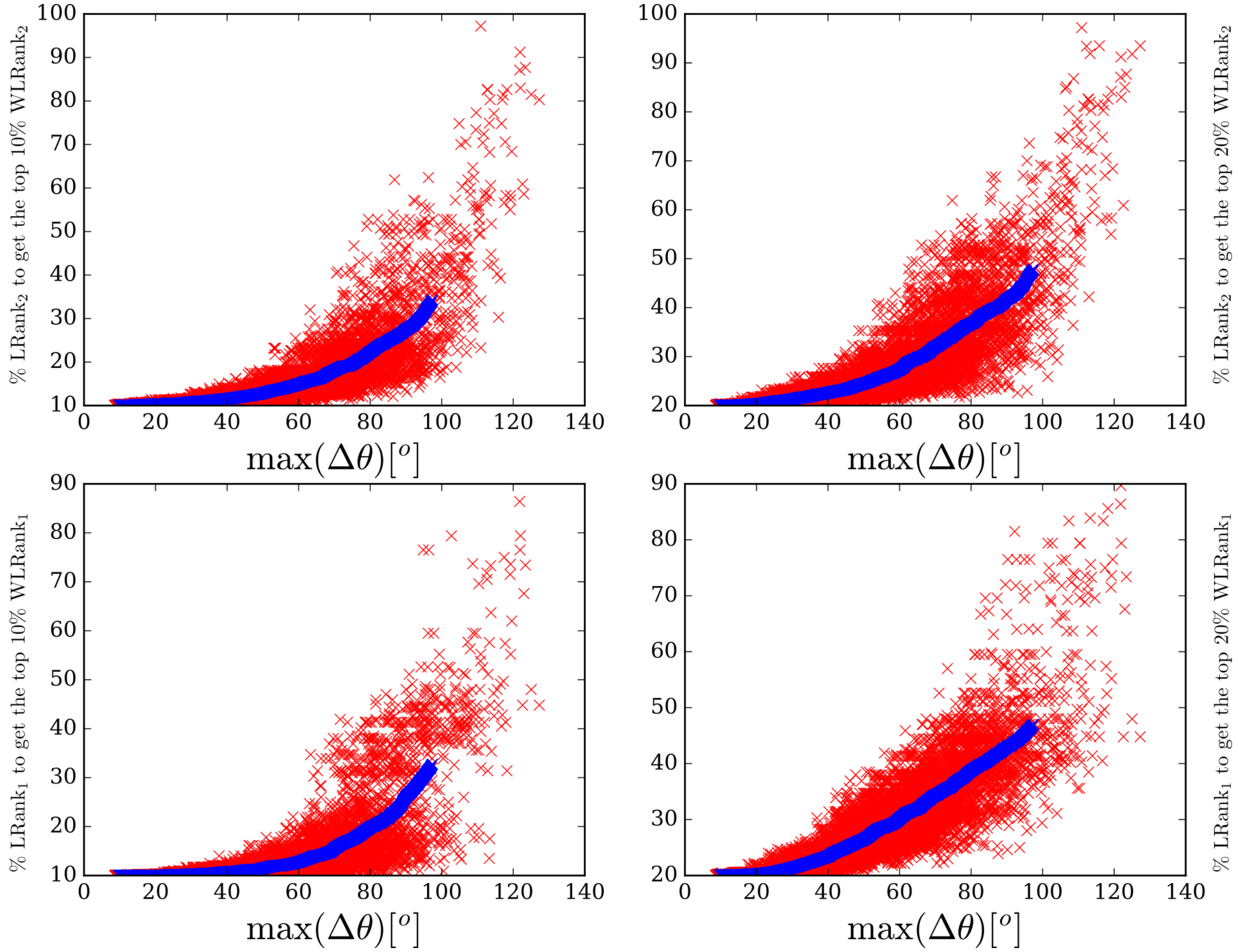

Fig. S3. Percentage of the nodes with highest LRank necessary to include the nodes with 10% and 20% highest WLRank. Percentage of the nodes with highest $\mathrm{LRank}_{1,2}$ necessary to give the top 10% (left), 20% (right) ranked nodes with $\mathrm{WLRank}_{1,2}$ for a random network of inertialess coupled oscillators with 1000 nodes obtained by rewiring with probability 0.5 a cyclic graph with constant nearest and next-to-nearest neighbor coupling (supplementary materials, materials and methods). Each of the 12000 red crosses corresponds to one of 1000 random natural frequency vector $\boldsymbol{P}^{(0)}$ with components randomly distributed in $[-0.5, 0.5]$ and summing to zero, multiplied by a prefactor $\beta = 0.4, 0.6, \dots 2.6$. The blue crosses correspond to running averages over 500 red crosses with consecutive values of $\max(\Delta\theta)$.
